# Editorial: Reward processing in motivational and affective disorders, volume II

**DOI:** 10.3389/fpsyg.2025.1549667

**Published:** 2025-04-17

**Authors:** Frank Ryan, Poornima Kumar, Nikolina Skandali, Brendan D. Adkinson

**Affiliations:** ^1^Imperial College London, London, United Kingdom; ^2^Center for Depression, Anxiety, and Stress Research, McLean Hospital and Department of Psychiatry, Harvard Medical School, Cambridge, MA, United States; ^3^Centre for Neuroimaging Sciences, Institute of Psychiatry, Psychology and Neuroscience, King's College London and Maudsley Hospital, South London and Maudsley NHS Foundation Trust, London, United Kingdom; ^4^Interdepartmental Neuroscience Program, Yale School of Medicine, New Haven, CT, United States

**Keywords:** reward processing, reinforcement learning, addiction—computational neuroscience, motivation, reward prediction error (RPE), cognitive behavioral therapy (CBT), affective neuroscience

As in the first Volume (Ryan and Skandali, 2016), we emphasize the dimensional or transdiagnostic role of reward processing in addictive disorders and associated mental health conditions. In the first volume, findings pointed to the intimate relationship between reward processing and cognition, apparent, for example, in both the remembrance of past rewards and the anticipation of future rewards (Dillon, 2015; Chekroud, 2015). In Volume 2, with the help of the expanded editorial team, the authors of this Editorial, we aim to build on these earlier efforts with a collection of four papers addressing the Research Topic from theoretical, experimental, and computational perspectives. All studies used computational or algorithmic approaches, with three in addition collecting data from human participants.

Before considering the new contributions to the Research Topic in more detail below, we believe it would be helpful to consider some important developments since the first volume was launched almost a decade ago. First, in clinical settings, new findings have shown that the promotion of positive affect by amplifying and elaborating reward anticipation and hedonic experience can match or even improve the clinical outcomes for depressed or anxious people compared to traditional cognitive behavior therapy (CBT) that targets negative affect (Craske et al., 2023). The Research Domain Criteria (RDoC, Insel et al., 2010) that created a conceptual basis for Volume 1 thus seems to be delivering on its promise to integrate science and practice using, in this case, the construct of reward processing spanning laboratory work and clinical practice. Furthermore, innovative findings in well-controlled and randomized clinical trials have provided a new avenue for pharmacological management of depression with new rapid-acting therapeutics, both ketamine/esketamine (Anand et al., 2023) and psychedelics (Carhart-Harris et al., 2021). Findings that drugs with hedonic properties such as these can prove therapeutic with emotional disorders such as depression resonate with the transdiagnostic, perspective on reward processing that predicates this Research Topic.

Additionally, computational models have accelerated our understanding of the complexity of reward processing and learning in healthy controls and individuals with mental health disorders, thereby providing insight into novel therapeutic interventions. This Research Topic has collated papers that have either proposed novel extensions of existing models or utilized sophisticated computational models to explore decision making and how cognitive processes such as working memory and attention can play a role in reward learning.

Turning to the work curated here, in the first paper Chase reconsiders key model parameters in reinforcement learning models and their implications for understanding individual differences in neural response to rewards. Through simulations, the author highlights the potential significance of lambda (the reinforcing efficacy of a particular reinforcer) over the more commonly studied alpha (learning rate) in determining reward prediction error (RPE)-related neural activity. Chase also introduced a derivative regressor to complement the standard RPE regressor in GLM models, which helps to capture unmodeled variation due to misspecification of the learning rate parameter. This study suggests that previous fMRI studies may have overlooked lambda's impact on observed relationships between clinical measures and RPE-related activations.

In the next study, Avila Chauvet and Mejía Cruz employed a dynamic Agent-Based Model (ABM), simulating reward sensitivity to gains and losses and the memory factor on the Iowa Gambling Task (IGT) specifically testing Bechara's hypotheses on decision-making. These researchers then compared the algorithmically generated outcomes with those from a sample of adults with substance abuse problems and unaffected controls. Although the number and characteristics of human participants limit the inferences that can be drawn, findings from both the human and agentic datasets point to the possibly modulatory role of working memory (WM) in reward learning. Accordingly, maintaining a recovery focused goal, or other rewarding long term goal in WM, could serve to increase anticipatory reward processing and thus align decision making with therapeutic objectives. This coheres with earlier findings (Bickel et al., 2011) showing WM training reduces delayed reward discounting in people with problematic stimulant use and can enable a significant reduction in alcohol consumption by problem drinkers (Houben et al., 2011). In sum, these convergent findings provide partial validation for psychotherapeutic approaches that enhance cognitive control by modifying attentional bias or by complementary approaches such as meditative practice.

Next, Peng et al. also used the IGT to explore the influence of social feedback on decision making with 192 participants recruited from the general population. Based on their analyses using the Outcome-Representation Learning (ORL) model, participants were found to form expectations for the effectiveness of feedback, in particular based on the (fictitious) past knowledge and experience of the advisory peer. Nevertheless, participants showed discriminatory learning by discounting the “expert” feedback when it was random, that is, ineffective. These findings suggest that implicit reward learning indexed by the IGT can be shaped by more transparent social learning mechanisms, such as feedback. Applied to clinical contexts, this hypothetically justifies the routine use of social reinforcement to guide decision-making for those people seeking to overcome addiction.

In the fourth and final study File et al. recruited 422 smokers, former smokers, and non-smokers via social media for an online experiment. They first measured attentional bias toward smoking-related stimuli, an index of salience or “wanting,” distinguished from “liking,” the latter seen as more reflective of hedonic processes according to Incentive Sensitization Theory (Robinson and Berridge, 2000). File et al. also attempted to capture and estimate the self-reported difference between “wanting” and “liking,” higher discrepancies being associated with higher levels of addiction (i.e., current smokers rather than former smokers in their cohorts) consistent with IST. The exploration of subjective reports of wanting and the willpower needed to suppress this is a notable innovation. Together with Peng et al., who applied social feedback to attempt to influence performance on the IGT, this promises to bring what are regarded as implicit cognitive and motivational processes within the range of introspection and thus therapeutic discourse.

We believe the new findings published here will stimulate and guide further research in this key area. We also hope the findings will focus clinicians' minds on the pivotal role played by reward processing in the genesis and remediation of motivational and emotional disorders.
